# Enabling DCIS subtyping: leveraging foundation models for robust grading and molecular biomarker scoring

**DOI:** 10.1038/s41523-026-00957-6

**Published:** 2026-05-06

**Authors:** S. Doyle, M. A. Oerlemans, J. Brunekreef, E. Marcus, L. Mulder, Y. Liu, I. Bouybayoune, E. J. Sawyer, A. M. Thompson, S. E. Pinder, Grand Challenge PRECISION Consortium, Jelle Wesseling, Alastair Thompson, Serena Nik-Zainal, Elinor J. Sawyer, Helen Davies, Andrew Futreal, Nicholas Navin, E. Shelley Hwang, Jos Jonkers, Jacco van Rheenen, Fariba Behbod, Esther H. Lips, Marjanka Schmidt, Lodewyk F. A. Wessels, Daniel Rea, Proteeti Bhattacharjee, Hilary Stobart, Deborah Collyar, Donna Pinto, Ellen Verschuur, Marja van Oirsouw, Jelle Wesseling, Alastair Thompson, Serena Nik-Zainal, Elinor J. Sawyer, Helen Davies, Andrew Futreal, Nicholas Navin, E. Shelley Hwang, Jos Jonkers, Jacco van Rheenen, Fariba Behbod, Esther H. Lips, Marjanka Schmidt, Lodewyk F. A. Wessels, Daniel Rea, Proteeti Bhattacharjee, Hilary Stobart, Deborah Collyar, Donna Pinto, Ellen Verschuur, Marja van Oirsouw, C. I. Sánchez, J. Teuwen, J. Wesseling, E. H. Lips

**Affiliations:** 1 Division of Radiation Oncology, Netherlands Cancer Institute, Amsterdam, the Netherlands; 2 Informatics Institute, University of Amsterdam, Amsterdam, the Netherlands; 3 Division of Molecular Pathology, Netherlands Cancer Institute, Amsterdam, the Netherlands; 4 School of Cancer & Pharmaceutical Sciences, King’s College London, London, UK; 5 Department of Surgery, Baylor College of Medicine, Houston, TX USA; 6 Department of Medical Imaging, Radboud University Nijmegen, Nijmegen, the Netherlands; 7 Department of Pathology, Netherlands Cancer Institute, Amsterdam, the Netherlands; 8 Department of Pathology, Leiden University Medical Center, Leiden, the Netherlands; 9 Netherlands Cancer Institute, Amsterdam, the Netherlands; 10 Baylor College of Medicine, Houston, TX USA; 11 University of Cambridge, Cambridge, UK; 12 King’s College London, London, UK; 13 MD Anderson Cancer Center, Houston, TX USA; 14 Duke University School of Medicine, Durham, NC USA; 15 Kansas University Medical Center, Kansas City, KS USA; 16 University of Birmingham, Birmingham, UK; 17 Independent Cancer Patients’ Voice, London, UK; 18 Patient Advocates in Research, New York, NY USA; 19 DCIS411, Houston, TX USA; 20 Borstkanker Vereniging Nederland, Utrecht, the Netherlands

**Keywords:** Cancer, Biomarkers, Medical research, Oncology, Cancer, Breast cancer, Cancer imaging

## Abstract

Ductal Carcinoma In Situ (DCIS) is a non-obligate precursor of invasive breast cancer. Due to a lack of reliable prognostic markers, nearly all women with DCIS undergo intensive treatment—often unnecessarily. The LORD trial addresses this by offering active surveillance to women with screen-detected, ER-positive, HER2-negative, grade 1 or 2 DCIS, aiming to reduce overtreatment. To support this, we developed a deep learning pipeline based on foundation models to predict grade, ER, and HER2 status directly from H&E-stained digitized pathology slides. Models were trained and tested on a Dutch multicenter dataset (n = 887) and externally validated on a UK dataset (n = 259). On the Dutch data, the models achieved mean AUROCs of 0.90 (ER), 0.84 (HER2), and 0.86 (grade); external validation yielded 0.80, 0.74, and 0.75, respectively. Using these outputs, we stratified patients according to active surveillance criteria, reaching balanced accuracies of 0.81 (Dutch) and 0.64 (UK), with corresponding NPVs of 0.86 and 0.76. Our models generalize across cohorts and reliably predict key biomarkers, supporting the identification of DCIS patients eligible for less aggressive management.

## Introduction

Ductal carcinoma in situ (DCIS) is a breast lesion frequently detected through population-based breast cancer screening, with the European standardized rate of 10.48 per 100.000 women for 2023. DCIS can progress to ipsilateral invasive breast cancer (iIBC), but at least 75% of DCIS lesions never progress or cause symptoms^1–3^. However, due to a lack of means to reliably distinguish non-progressive from potentially progressive DCIS, almost all women with DCIS are treated similarly to those with early stage invasive breast cancer (IBC). Depending on tumor and breast size, and patients’ preferences, this treatment typically includes breast-conserving surgery followed by radiotherapy or mastectomy^4^. Consequently, women with non-progressive DCIS carry the burden of overtreatment.

In pathology, DCIS is classified using a three-tier grading system, i.e., grade 1 (well differentiated), grade 2 (intermediately differentiated) and grade 3 (poorly differentiated), mainly based on the degree of nuclear atypia^5^. Ongoing trials, including COMET and LORD^6,7^, are investigating whether active surveillance is a safe alternative for women with screen-detected, grade 1 or 2 DCIS. There is some evidence for a link between higher grades and increased recurrence risk^8,9^, but it is unclear whether this applies only to recurrence as ipsilateral DCIS or also to iIBC. Additionally, grading is subject to inter-observer variability^5,10^.

Besides grade, the expression of human epidermal growth factor receptor 2 (HER2) and estrogen receptor (ER) may help characterize DCIS. These markers are routinely measured via immunohistochemistry (IHC) for molecular subtyping in IBC but not yet in DCIS. If HER2 is positive, it is associated with higher grade and larger lesion size^11–13^, and may help to refine grading. It is potentially linked to an elevated risk of recurrence in DCIS^8,12–16^ and lack of ER expression^12^. In contrast, ER-positive DCIS is typically lower grade and less aggressive^17,18^. However, ER status does not predict local recurrence risk^8,17,19^. Similarly to HER2-positive DCIS, this finding may be confounded by factors related to more intensive treatment in ER-negative DCIS lesions. Currently, these markers, alongside grade, are used in the LORD and COMET trials to check the eligibility of women that might be included in these trials.

The LORD trial has shown that the majority of women with lower-grade DCIS opt for active surveillance over conventional treatment when presented with the choice^6^, underscoring the need for reliable tools to identify low-risk cases to support safe and informed decision-making. Currently, determining ER and HER2 status requires additional tissue slices, IHC stainings, and pathologist assessment, making it a costly and time-consuming process. Deep learning models applied to H&E-stained whole slide images (WSIs) could streamline this workflow by reducing variability from pathology assessments, improving efficiency, and lowering costs. Deep learning models have already demonstrated expert-level performance in predicting DCIS grade^20^, and have achieved robust results in predicting ER status and, to a lesser extent, HER2 status, in invasive breast cancer^21–24^. Some of these studies have employed the recently popularized foundation models, which leverage large, diverse histopathological datasets to further enhance robustness, scalability, and overall performance^25–29^.

In this study, we predicted ER status, HER2 status, and grade from H&E-stained WSIs of primary, pure DCIS lesions using a deep learning pipeline that includes a foundation model encoder. Our models were trained and evaluated on a large internal dataset (*n* = 935) and externally validated on an independent dataset (*n* = 276) acquired with a different scanner.

## Results

In this study, we developed deep learning models to predict the biomarkers ER, HER2, and grade in DCIS directly from H&E-stained WSIs, trained and tested on the Dutch dataset and externally validated on the UK-based Sloane dataset (see Table 1 for cohorts). We assessed model performance for each biomarker individually using optimized thresholds and combined model outputs to predict active surveillance trial inclusion parameters,’ where patients predicted as lower-risk (ER-positive, HER2-negative, and grade 1 or 2) are considered eligible for active surveillance in the LORD trial.

**Table 1:** Patient characteristics of the Dutch and Sloane external test cohort with fraction of total in brackets if applicable

Patient characteristics	Precision_NKI_89_05	Sloane
Total patients	935	260
Age patient mean	57.6	59.6
Age Patient std	10.6	6.4
FFPE block age mean	26.8	
FFPE block age std	3.8	
GRADE 1	132 (0.14)	31 (0.12)
GRADE 2	479 (0.51)	78 (0.3)
GRADE 3	276 (0.3)	150 (0.58)
GRADE missing	48 (0.05)	1 (0.0)
HER2 negative	564 (0.61)	144 (0.55)
HER2 positive	222 (0.24)	74 (0.28)
HER2 missing	149 (0.16)	42 (0.16)
ER negative	155 (0.17)	64 (0.25)
ER positive	642 (0.69)	182 (0.70)
ER missing	138 (0.15)	14 (0.05)

### Stable biomarker predictions on the Dutch dataset

We first assessed the performance of our biomarker models, derived from each outer fold of the nested cross-validation, on the Dutch dataset, as shown in Table 2 and Supplementary Fig. 2. The binarization thresholds applied to the biomarker predictions are provided in Supplementary Table 3. For ER prediction, the models achieved an AUROC of 0.90 (±0.06) and a balanced accuracy of 0.83 (±0.04). For HER2, the AUROC was 0.84 (±0.03), with a balanced accuracy of 0.75 (±0.03). For high-grade prediction, the models achieved an AUROC of 0.86 (±0.02) and a balanced accuracy of 0.79 (±0.02). The low standard deviations and minimal variability in ROC curves across folds indicate stable biomarker predictions.

**Table 2:** Performance metrics of biomarker prediction models evaluated on the Dutch and Sloane datasets

Variable	Balanced accuracy	F1	Specificity	Sensitivity	PPV	NPV	AUROC
Dutch dataset							
ER	0.83 (±0.04)	0.88 (±0.02)	0.84 (±0.07)	0.83 (±0.03)	0.95 (±0.03)	0.54 (±0.05)	0.90 (±0.06)
HER2	0.75 (±0.03)	0.64 (±0.04)	0.81 (±0.06)	0.70 (±0.05)	0.60 (±0.06)	0.87 (±0.01)	0.84 (±0.03)
High grade	0.79 (±0.02)	0.69 (±0.04)	0.78 (±0.06)	0.79 (±0.03)	0.62 (±0.08)	0.89 (±0.02)	0.86 (±0.02)
Sloane dataset							
ER	0.71 (±0.05)	0.71 (±0.14)	0.81 (±0.09)	0.61 (±0.18)	0.90 (±0.02)	0.44 (±0.07)	0.80 (±0.02)
HER2	0.62 (±0.03)	0.57 (±0.02)	0.31 (±0.09)	0.93 (±0.06)	0.41 (±0.02)	0.91 (±0.06)	0.74 (±0.01)
High grade	0.68 (±0.03)	0.74 (±0.05)	0.60 (±0.13)	0.76 (±0.10)	0.73 (±0.04)	0.66 (±0.07)	0.75 (±0.02)

Values represent the mean and standard deviation (in brackets) across outer cross-validation folds.

*NPV* negative predictive value, *PPV* positive predictive value, *AUROC* area under the receiver operating characteristic curve.

### Biomarker models generalize with low variability on external dataset

**Fig. 1:**
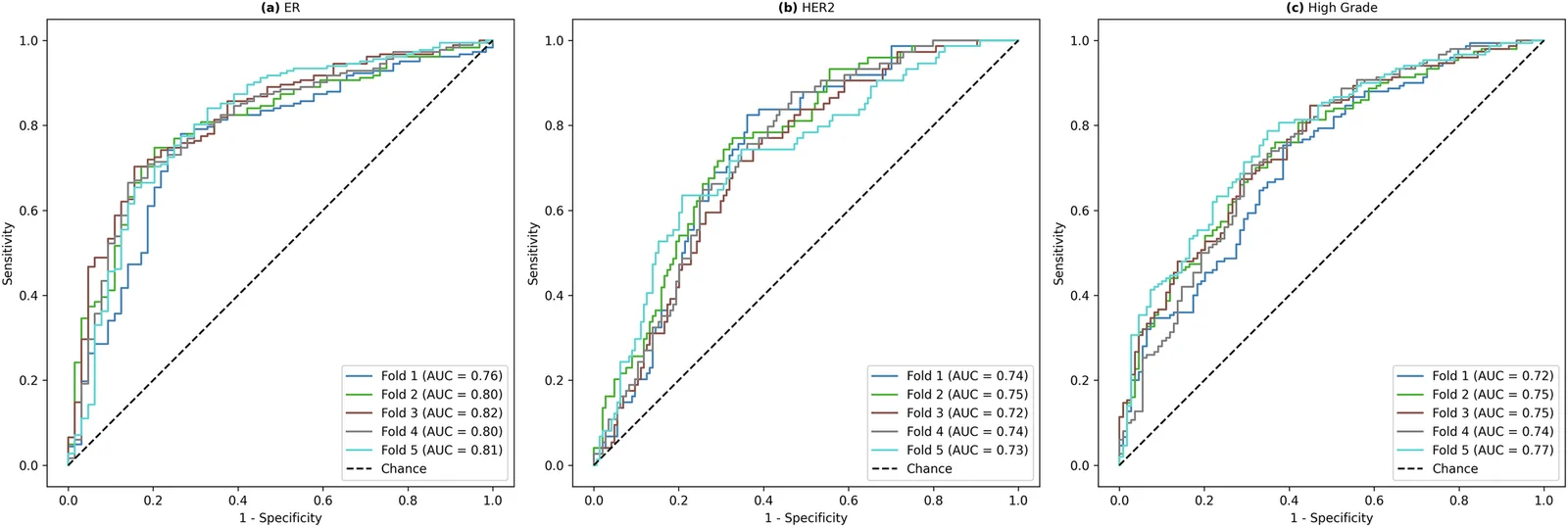
Receiver operating characteristic (ROC) curves for predictions on the external Sloane dataset. ROC curves from each model across outer cross-validation folds are shown. **a** ROC curves for ER status, with a mean AUROC of 0.80 ( ± 0.02); **b** ROC curves for HER2 status, with a mean AUROC of 0.73 (±0.01); and **c** ROC curves for grade, with a mean AUROC of 0.75 (±0.02).

We further evaluated our biomarker models on the external dataset. For ER status, the AUROC was 0.80 (±0.02) and the balanced accuracy was 0.71 (±0.05). For HER2 status, the AUROC was 0.74 (±0.01) and the balanced accuracy was 0.62 (±0.03). Additionally, the HER2 models showed an increased propensity to predict positives on the external dataset, with specificity of 0.31 (±0.10) and a sensitivity of 0.93 (±0.06). For high-grade prediction, the AUROC was 0.75 (±0.02) and the balanced accuracy was 0.68 (±0.03). For all biomarkers, the models from different folds exhibited low variability in performance on the external dataset, with even less variability than the internal dataset, as indicated by the closely aligned ROC curves in Fig. 1 and the low standard deviations in Table 2.

### Prediction of active surveillance trial criteria by combining model outputs

We also evaluated our models’ ability to identify patients eligible for active surveillance under the LORD trial criteria. Table 3 presents the results using binarized predictions from the individual biomarker models. In the Dutch dataset, the models achieved a balanced accuracy of 0.81 (±0.02), an NPV of 0.86 (±0.04), with a specificity of 0.79 (±0.03). Table 4 shows that on average 77.9 (±6.7) patients could be included in the LORD trial, 11.2 (±2.8) were misclassified, and 17.7 (±1.9) were missed.

**Table 3:** Performance metrics for predicting the patients fit for active surveillance evaluated on the Dutch and Sloane datasets

Dataset	Balanced accuracy	F1	Specificity	Sensitivity	PPV	NPV
Dutch	0.81 (±0.02)	0.79 (±0.02)	0.79 (±0.03)	0.83 (±0.04)	0.75 (±0.02)	0.86 (±0.04)
Sloane	0.64 (±0.03)	0.83 (±0.01)	0.33 (±0.10)	0.94 (±0.03)	0.74 (±0.02)	0.76 (±0.07)

Values represent the mean and standard deviation (in brackets) across outer cross-validation folds.

*NPV* negative predictive value, *PPV* positive predictive value.

**Table 4:** Predicted LORD-groups in the Dutch and Sloane dataset

Dataset	#Predicted LORD-positive	#Predicted LORD-negative	#Misclassified LORD-positive	#Misclassified LORD-negative
Dutch	70.7 (±4.4)	77.9 (±6.7)	17.7 (±1.9)	11.2 (±2.8)
Sloane	180.0 (±10.9)	31.0 (±10.9)	46.3 (±6.5)	8.2 (±4.6)

Total predicted patients, and misclassified patients per LORD group are shown. Model predictions are presented as means over the outer folds, with standard deviations in brackets.

In the external dataset, the balanced accuracy was 0.64 (±0.03), with an NPV of 0.76 (±0.07) and a specificity of 0.33 (±0.10). The low specificity in the external dataset reflects the model’s conservative approach to predicting the negative class, which is also reflected in its high sensitivity of 0.94 (±0.03). Table 4 shows that, for the Sloane dataset, on average 31.0 (±10.9) patients could be included in the LORD trial, while 8.2 (±4.6) were misclassified, and 46.3 (±6.5) were missed.

## Discussion

Our study demonstrates the potential of using deep learning models in predicting the status of ER and HER2 expression, as well as DCIS grade from WSIs. This could eliminate the need for labor-intensive IHC staining and pathology review. Our models, which incorporated a frozen foundation model encoder, achieved robust biomarker predictions when tested internally on a comprehensive Dutch national cohort and externally with the UK-based Sloane cohort. Specifically, the models yielded AUROCs of 0.90 and 0.80 for ER on the Dutch and external datasets, respectively, and 0.84 and 0.73 for HER2 and 0.86 and 0.75 for grade.

Previous studies have predicted IHC markers from WSIs, mainly in invasive breast cancer. Filiot et al. reported ER AUROCs up to 0.88 and HER2 AUROCs up to 0.74 using a similar methodology to ours^22^. In contrast, Shamai et al. and Farahmand et al. achieved higher AUROCs (0.93–0.96 for ER and 0.69–0.87 for HER2) by incorporating tumor area labeling^23,24^, with Farahmand et al. noting an 8% improvement in HER2 prediction from the labels. Our external AUROCs for ER and HER2 align with those of Filiot et al., indicating that tumor annotations may further enhance performance.

However, our models used tile embeddings generated by a foundation model, which may reduce the reliance on explicit tumor annotations. During training, the models learned to attend to tumor regions, as illustrated by WSI visualizations showing strong alignment between model-attended areas and DCIS regions (Supplementary Figs. 3–5). Additionally, Valieris et al. distinguished HER2-negative (IHC 0), HER2-low (IHC 1+ or 2+), and HER2-high (IHC 3+) cases with a model that encoded tiles using the UNI foundation model^26^, reported comparable performance when restricting input to tumor-enriched regions compared to using full-slide images^30^. While HER2 classification with three categories has emerging relevance in invasive breast cancer, our binary approach for DCIS aligns with active surveillance trial inclusion criteria.

The consistently higher performance of ER models compared to HER2 models suggests that predicting HER2 status from WSIs may be inherently more difficult or not consistently feasible. Further research is needed to identify which histopathological features enable accurate HER2 prediction from WSIs. Explainability techniques may help uncover these features, as shown by Valieris et al.^30^, who used CLAM, an attention-based multiple instance learning model with clustering constraints, to identify high-attention tiles. Applying PCA to these tiles revealed clustering by HER2 class, and visual inspection of representative tiles of the clusters showed that HER2-positive tiles often displayed apocrine features, including higher nuclear grade and infiltrative growth, whereas HER2-low tiles were enriched for chronic inflammation, fibrotic tissue, and lower nuclear grades. These findings highlight the potential of explainability-driven models to improve both interpretability and classification performance.

Wetstein et al. showed that deep learning models could predict DCIS grade with slightly higher inter-observer agreement compared to three reviewers^20^. Additionally, Huang et al. reported an AUROC of 0.97 on a held-out internal test set for nuclear grading of DCIS^31^, compared to our mean AUROC of 0.86. In our study, the grading system incorporated additional features beyond nuclear grade, which may have introduced greater ambiguity in the ground truth labels. Additionally, three pathologists, rather than two in Huang et al., collaborated to determine the ground truth labels, which could have contributed to label ambiguity, despite reducing the potential for inter-observer variability. Huang’s study also differed from ours in terms of patient characteristics, including a higher proportion of high-grade DCIS (54% vs. 36% in our dataset) and fewer -hard to assess- grade 2 cases (32% vs. 51%), along with inclusion of atypical ductal hyperplasia (10%) and DCIS with microinvasion (4%).

Using the biomarker predictions, we identified women with DCIS who would be eligible for active surveillance under the LORD trial criteria, achieving NPVs above 0.79. On the external dataset, however, specificity was low, likely due to the limited specificity of the HER2 model on this dataset. As thresholds are not optimized on the external set, additional calibration of thresholds on external sets is necessary to guarantee high performance.

In the presence of class imbalance, which is present in our dataset for ER (ER-negative: 14% in the Dutch dataset; 30% in Sloane dataset), HER2 (HER2-positive: 30% in the Dutch dataset; 42% in Sloane dataset) and grade (grade 3: 30% in Dutch dataset; 57% in Sloane dataset), a model may become biased toward the majority class. To address this, we optimized biomarker classification thresholds using balanced accuracy, a metric that accounts for both sensitivity and specificity and ensures an equal consideration of both classes. The optimized thresholds generally resulted in improved balanced accuracy compared to the commonly used threshold of 0.5 (Supplementary Tables 4 and 5). An exception was HER2 prediction, where the lower, optimized threshold resulted in reduced external performance. However, this was likely due to the model’s tendency to generate high prediction scores for HER2, combined with greater overlap between true positive and true negative scores in the external dataset. This finding highlights the need for further classification refinement, particularly in improving the calibration and discrimination of classes across dataset in the HER2 models.

The accurate prediction of ER status both in our DCIS datasets and in invasive breast cancer datasets, suggests that there are consistent ER-related signals in WSIs that deep learning models detect. This highlights the models’ potential for clinical integration. In clinical practice, ER status is routinely assessed for breast cancer subtyping and treatment decisions. In some countries, such as the U.S., ER status is furthermore used to identify candidates for endocrine therapy among women diagnosed with DCIS^17^.

Routine ER prediction could also help clarify its role in DCIS. While ER expression is negatively associated with aggressive features like microinvasion^18^ and higher histological grade^17^, it is not strongly predictive of invasive recurrence risk^8,17,19^. Similarly, routine HER2 assessment in DCIS could provide insights into its association with in-situ and invasive recurrence^12,14^, as well as its role in the DCIS-to-IBC transition^32,33^. Another opportunity lies in exploring the biological mechanisms underlying the notably higher frequency of HER2 overexpression in DCIS compared to IBC^34,35^ and the association between HER2-positive DCIS and immune microenvironmental features, such as tumor-infiltrating lymphocytes^36^.

The clinical utility of DCIS grading, which is based on histological features primarily reflecting nuclear atypia, is limited by interobserver variability, lesion heterogeneity, and a weak association with the risk of invasive recurrence. A robust AI model could address these issues improving diagnostic consistency. Furthermore, given the correlation between grade, ER, and HER2 status, routine automated prediction of ER and HER2 could refine grading^5^.

In settings where clinical treatment decisions rely on the combined output of multiple biomarkers, such as the active surveillance trials, the classification of each individual biomarker within the AI-based tool must be exceptionally robust and accurate, as errors can accumulate and undermine the overall risk assessment.

Our study demonstrates the potential of deep learning models for the automated assessment of ER, HER2, and grade in DCIS. However, several limitations remain. While we validated our models on one external dataset using a different scanner type, broader validation on larger, diverse external datasets is necessary before clinical implementation. Additionally, HER2 and grade prediction performance should be further optimized, potentially by leveraging explainable AI approaches, such as incorporating tumor-region annotations.

While the distribution of assessed biomarkers in our dataset was comparable to that of the broader DCIS population, the cohort was enriched for patients who developed invasive recurrence. This enrichment may have influenced the types of histopathological features presented to the model, potentially affecting the complexity of the biomarker prediction task. Furthermore, validation on biopsies could enhance the clinical applicability of the presented models.

Given the high interobserver variability in grade evaluation and the fact that ER and HER2 are not routinely assessed in DCIS, AI-driven predictions offer a more consistent and accessible alternative to manual scoring. Future studies should validate models like ours on large external cohorts and explore their integration into clinical practice, where their use would produce data to support further research and evaluation of clinical utility. Additionally, further research may focus on model explainability, enhancing both performance and interpretability while advancing our understanding of ER, HER2, and pathological characteristics in DCIS progression.

## Methods

### Data and study design

In our study, we used two cohorts, referred to as the Dutch and Sloane cohorts. The inclusion criteria were a primary, pure DCIS diagnosis and the availability of tissue resections. Detailed descriptions of the cohorts are provided in Table 1. This retrospective study was conducted in accordance with the Declaration of Helsinki. For the Dutch cohort, ethical approval was granted by the Institutional Review Board of the Netherlands Cancer Institute - Antoni van Leeuwenhoek (IRBd22-110), which waived the need for informed consent due to the retrospective nature of the study. Secondary data usage adhered to Dutch regulations and the Federa-COREON Code of Conduct under an opt-out regime. Data access was approved by the Netherlands Cancer Registry (K12.281) and PALGA (LZV990). The Sloane cohort, part of the United Kingdom National Health Service Breast Screening Programme, was approved by the UK Health Research Authority (ethical approval 19/LO/0648). Informed consent was obtained for the Sloane cohort.

From a Dutch nationwide cohort of 10,090 women diagnosed between 1989 and 2005 with follow-up until 2019^9^, slides of good quality with confirmed pure DCIS were included in the case-control series used by Visser et al.^14^ and the case-cohort series used by Groen et al.^37^ (Supplementary Fig. 1). From these series, we selected 935 patients who met our inclusion criteria. Among these, 132 patients missed data for ER status, 149 patients missed data for HER2 status and 48 patients were missing data for grade. In total, 192 patients had data missing for one or more variables. Each WSI was manually reviewed for the exclusive presence of DCIS, and grading was performed and revised by three expert pathologists (J. Wesseling, E.J. Groen, and K. van de Vijver). They assessed slides on morphologic characteristics, including DCIS architecture and nuclear grade, the presence of calcifications and necrosis, and microenvironmental characteristics like stromal features and the presence of lymphocytes. Their interrater variability was judged to be within expected ranges.

The Sloane prospective cohort, which comprises 33% of DCIS patients diagnosed within the UK National Health Service Breast Screening Programme between 2003 and 2012^38^, a selection of screen-detected pure DCIS cases with clear surgical margins >1 mm was made. We included a subset of 260 patient samples that fulfilled our inclusion criteria. Within this subset, 14 patients missed data for ER status, 42 for HER2 and 1 for grade, while 211 patients had complete clinicopathological variables.

### Data preparation

Formalin-fixed paraffin-embedded tissue sections of DCIS lesions were stained with H&E. They were scanned at ×20 magnification. In the Dutch cohort a Leica Aperio AT2 scanner was used, and a Hamamatsu Nanozoomer 2.0-HT scanner was used for the Sloane cohort. We produced tissue masks for the WSIs using a U-Net segmentation model which was trained in-house. Following previously described procedures^25^, we tiled WSIs into non-overlapping patches of 224 × 224 pixels at a resolution of 0·5 μm/pixel, providing clear views of small-sized ducts and individual cells. Finally, we removed patches without segmented tissue.

For training and assessment, we binarized variables as follows: HER2 (negative = IHC 0, 1 + , 2 + ; positive = IHC 3 + ), grade (negative = grade 1, 2; positive = grade 3), and ER (negative = IHC 0-9%; positive = IHC 10-100%). To validate the HER2 classification, we performed CISH on 20 DCIS lesions with IHC 2+ staining; all cases were non-amplified, confirming that IHC 2+ typically corresponds to non-amplified HER2 in DCIS. In the Sloane cohort, HER2 status was determined through in-situ hybridization rather than immunohistochemistry, as was the case for the Dutch Cohort.

We also assessed a model for active surveillance trial inclusion, and used the LORD inclusion criteria: the combination of the following three biomarkers qualifies as LORD-inclusion criteria: ER-positive, HER2-negative, grade 1 or 2).

### Model, training, and assessment

We evenly split the Dutch dataset into five outer folds, ensuring a similar distribution of covariates across folds (SupplementaryTable 1). For each outer fold, we performed an inner cross-validation (*k* = 5) using GroupStratifiedKFold (scikit-learn v.1.3.2^39^), stratifying by the target biomarker and grouping by tissue block ID to ensure that all tiles from a patient’s WSI were assigned to the same fold.

**Fig. 2:**
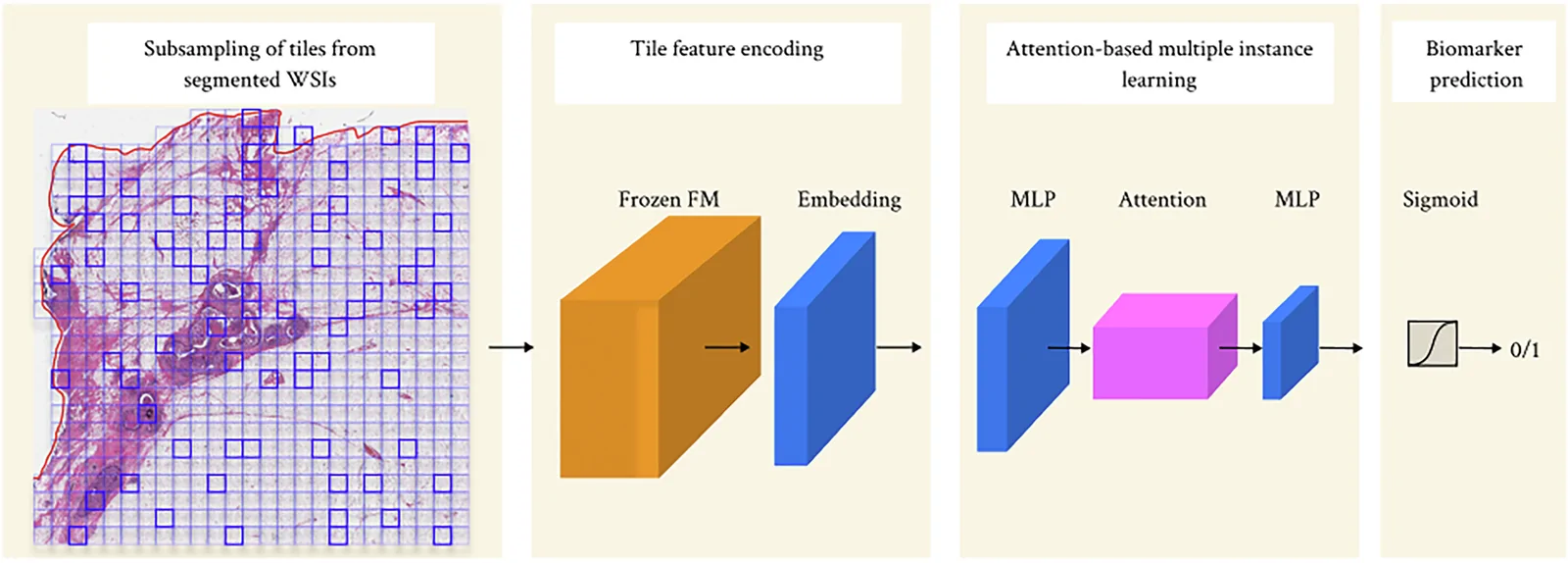
Pipeline of the DCIS biomarker prediction models. Whole slide images (WSIs) are segmented and tiled, with 1000 tiles randomly subsampled from each WSI. The tiles are processed through the frozen foundation model encoder, “Phikon” (Filiot et al.^25^). The resulting features are then passed to a multi-layer perceptron (MLP) for target biomarker prediction. During training, the MLP is optimized using attention-based multiple instance learning (AB-MIL)^40^ to account for tile importance.

Figure 2 illustrates the architecture and pipeline we used for training our DCIS biomarker prediction models. The pipeline consists of a frozen encoder and a classifier. The encoder is the Phikon model, an open-source Vision Transformer Base (ViT-B) trained on 40 million pan-cancer histology tiles from TCGA using the iBOT framework^25^. The classifier is a two-layer multi-layer perceptron (MLP) trained using attention-based multiple instance learning (AB-MIL)^40^. In this framework, each WSI is represented as a set of tiles, and the model learns to assign varying levels of importance (attention weights) to individual tiles when aggregating them into the WSI prediction. To train the classifier, we used the methods described in Filiot et al.^27^. We performed a grid search to tune the hyperparameters of our models with the AUROC as the evaluation metric. We present further details on model configurations in supplemental Table 2. We trained models on an NVIDIA RTX A6000 GPU with 48GB of memory.

For each fold of the outer cross-validation, we constructed an ensemble model using the best-performing checkpoints from the corresponding inner cross-validation folds. We selected optimal checkpoints based on the area under the receiver operating characteristic curve (AUROC), calculated using the “torchmetrics” library (v1.6.1)^33^. We assessed the performance of these ensemble models for clinical biomarkers individually, and for active surveillance trial inclusion. As evaluation metrics, we chose, next to the AUROC, the balanced accuracy, F1-score, positive predictive value (PPV), negative predictive value (NPV), sensitivity, and specificity, which are based on binarised predictions. We optimized the threshold for binarization in trained models for maximum balanced accuracy to ensure equal consideration of both positive and negative classes, addressing class imbalance. We defined the binarization threshold for each ensemble model as the average of the optimized thresholds derived from the individual models within the ensemble. To evaluate the active surveillance trial inclusions prediction, we used outputs from the individual biomarker models. We assessed the group prediction based on binary predictions for each biomarker.

## Supplementary information

Supplementary Information

## Data Availability

The data used in this study were shared among members of the PRECISION Consortium under a unified funding initiative. Requests for access to the data may be accommodated upon reasonable inquiry directed to the corresponding author, subject to appropriate agreements. The underlying code for this study is not publicly available but may be made available to qualified researchers on reasonable request from the corresponding author.

## Code availability

The underlying code for this study is publicly available at https://github.com/NKI-AI/dcis-biomarkers.
