# Supplementary material for: Enabling DCIS subtyping: leveraging foundation models for robust grading and molecular biomarker scoring
Source: NPJ Breast Cancer. 2026 May 6;12:124. doi: 10.1038/s41523-026-00957-6 (PMC13623920; doi:10.1038/s41523-026-00957-6)
Supplement: Supplementary file 1 [file 41523_2026_957_MOESM1_ESM.docx]

# **Supplement**

## **Supplemental figures**

**Supplemental figure 1.** Flowchart of patient inclusion. Dutch dataset (left); Sloane dataset (right).

**Supplemental figure 2.** ROC curves on the Dutch dataset. Each line denotes an ensemble model trained and evaluated on a different outer fold of the data. (a) denotes predictions for estrogen receptor (ER) status with a mean AUROC of 0.90. (b) denotes predictions for HER2 receptor status with a mean AUROC of 0.84 and (c) denotes predictions for DCIS grade 1 and 2 versus DCIS grade 3 with a mean AUROC of 0.86.

|  |  |  |  |
| --- | --- | --- | --- |
|  |  |  |  |
|  |  |  |  |

**Supplemental figure 3**: Attention values visualized on the slide for a model predicting HER2 with digitized HER2 staining in the last column. More yellow means more attention to that particular patch. We expect the model to look at neoplastic ducts as HER2 is a receptor that presents itself on epithelial cells in these ducts, and the attention maps show a similar pattern.

|  |  |  |  |
| --- | --- | --- | --- |
|  |  |  |  |
|  |  |  |  |

**Supplemental figure 4**: Attention values visualized on the slide for a model predicting ER with digitized ER staining in the last column. More yellow means more attention to that particular patch. We expect the model to look at neoplastic ducts as ER is a receptor that presents itself on epithelial cells in these ducts, and the attention maps show a similar pattern.

|  |  |  |
| --- | --- | --- |
|  |  |  |
|  |  |  |

**Supplemental figure 5** Attention values visualized on the slide for a model predicting grade. More yellow means more attention to that particular patch. We expect the model to look at ducts as grade is determined by pathologists by looking at properties of the cells within the duct, and the attention maps show a similar pattern.

## **Supplemental tables**

| **Patient Characteristics** | **Outer train folds means** | **Outer train folds 95% CIs** | **Outer validation folds means** | **Outer validation folds 95% CIs** |
| --- | --- | --- | --- | --- |
| Age Patient mean | 57.6 | (57.4, 57.8) | 57.6 | (56.8, 58.4) |
| Age Patient std | 10.6 | (10.5, 10.7) | 10.6 | (10.1, 11.1) |
| FFPE block age mean | 26.8 | (26.7, 26.8) | 26.8 | (26.5, 27.0) |
| FFPE block age std | 3.8 | (3.8, 3.9) | 3.8 | (3.7, 4.0) |
| GRADE 1 | 0.14 | (0.13, 0.15) | 0.14 | (0.10, 0.19) |
| GRADE 2 | 0.51 | (0.50, 0.52) | 0.51 | (0.48, 0.55) |
| GRADE 3 | 0.29 | (0.29, 0.30) | 0.30 | (0.26, 0.33) |
| GRADE Missing | 0.05 | (0.05, 0.05) | 0.05 | (0.04, 0.06) |
| HER2 Negative | 0.61 | (0.59, 0.61) | 0.61 | (0.56, 0.64) |
| HER2 Positive | 0.24 | (0.23, 0.24) | 0.24 | (0.22, 0.25) |
| HER2 Missing | 0.16 | (0.15, 0.17) | 0.16 | (0.12, 0.20) |
| ER Negative | 0.17 | (0.16, 0.18) | 0.17 | (0.14, 0.19) |
| ER Positive | 0.69 | (0.67, 0.70) | 0.69 | (0.63, 0.74) |
| ER Missing | 0.15 | (0.14, 0.16) | 0.15 | (0.12, 0.18) |

**Supplemental table 1.** Patient characteristics as absolute numbers or fractions of the outer folds/splits of the nested cross-validation in the Dutch dataset.

| Parameter | Value | Notes |
| --- | --- | --- |
| Optimizer | Adam |  |
| Learning rate | 1e-4 | 1e-5 for grade experiments |
| Weight decay | 0 |  |
| Dropout | 0.1 |  |
| Before attention hidden layer size | 128 |  |
| Classifier hidden layer size | [128, 64] |  |
| Attention dimension | 128 |  |
| Batch size | 16 |  |
| Tiles per sample | 1000 | As described in Filiot et al. (27) |
| Tile size | 224 x 224 | As described in Filiot et al. (27) |
| Microns per pixel | 0.5 | As described in Filiot et al. (27) |
| Loss | Cross entropy loss |  |
| Checkpoint selection metrics | AUROC |  |
| Number of outer folds | 5 |  |
| Number of inner folds | 5 |  |

**Supplemental table 2.** Parameters used for training the deep learning pipeline

| Variable | Outerfold 0 | Outerfold 1 | Outerfold 2 | Outerfold 3 | Outerfold 4 |
| --- | --- | --- | --- | --- | --- |
| ER | 0.79 (±0.13) | 0.78 (±0.07) | 0.70 (±0.16) | 0.70 (±0.18) | 0.88 (±0.03) |
| HER2 | 0.33 (±0.17) | 0.35 (±0.19) | 0.34 (±0.15) | 0.37 (±0.06) | 0.40 (±0.19) |
| Grade | 0.36 (±0.13) | 0.30 (±0.10) | 0.27 (±0.11) | 0.40 (±0.02) | 0.29 (±0.07) |

**Supplemental table 3:** Optimized thresholds per outerfold. We optimize the thresholds per innerfold and show the mean threshold here. Standard deviation over the innerfolds between brackets.

| Variable | Balanced Accuracy | F1 | Specificity | Sensitivity | PPV | NPV |
| --- | --- | --- | --- | --- | --- | --- |
| ER | 0.77 (±0.06) | 0.92 (±0.02) | 0.60 (±0.12) | 0.94 (±0.02) | 0.90 (±0.04) | 0.71 (±0.06) |
| HER2 | 0.74 (±0.03) | 0.62  (±0.05) | 0.88 (±0.04) | 0.59 (±0.06) | 0.67 (±0.07) | 0.85 (±0.01) |
| High grade | 0.73 (±0.04) | 0.62 (±0.06) | 0.90 (±0.03) | 0.55 (±0.09) | 0.72 (±0.04) | 0.82 (±0.03) |
| Active surveillance stratification | 0.78 (±0.01) | 0.73 (±0.02) | 0.91 (±0.01) | 0.64 (±0.02) | 0.85 (±0.02) | 0.77 (±0.02) |

**Supplemental table 4.** Different metrics evaluated on the Dutch test set for predicting the biomarkers. The metrics are calculated at the threshold of 0.5, the average over the folds is shown with the standard deviation between brackets.

| Variable | Balanced Accuracy | F1 | Specificity | Sensitivity | PPV | NPV |
| --- | --- | --- | --- | --- | --- | --- |
| ER | 0.68 (±0.04) | 0.86 (±0.01) | 0.46 (±0.10) | 0.90 (±0.04) | 0.83 (±0.02) | 0.61 (±0.05) |
| HER2 | 0.67 (±0.02) | 0.60 (±0.02) | 0.53 (±0.09) | 0.82 (±0.07) | 0.47 (±0.03) | 0.85 (±0.03) |
| High grade | 0.64 (±0.02) | 0.56 (±0.04) | 0.85 (±0.05) | 0.43 (±0.06) | 0.80 (±0.04) | 0.52 (±0.02) |
| Active surveillance stratification | 0.69 (±0.02) | 0.78 (±0.03) | 0.62 (±0.07) | 0.76 (±0.06) | 0.81 (±0.02) | 0.56 (±0.05) |

**Supplemental table 5.** Different metrics evaluated on the Sloane external set for predicting the biomarkers. The metrics are calculated at the threshold of 0.5, the average over the folds is shown with the standard deviation between brackets.
